# Supplementary material for: Hypoxia Modulates Transmembrane Prostatic Acid Phosphatase (TM-PAP) in MCF-7 Breast Cancer Cells
Source: Int J Mol Sci. 2025 Feb 23;26(5):1918. doi: 10.3390/ijms26051918 (PMC11900489; doi:10.3390/ijms26051918)
Supplement: Supplementary file 1 [file ijms-26-01918-s001.zip › ijms-3456925-supplementary.pdf]

Table S1. Predicted phosphorylation sites with scores ≥0.50 and specific for PKC in the primary sequences of TM-PAP.

**Amino acid**	**Score**
Ser^{96}	0.631
Thr^{107}	0.503
Thr^{190}	0.664
Thr^{203}	0.513
Ser^{279}	0.835

Analysis of TM-PAP (RefSeq: NP_001127666) using NetPhosK 3.1 prediction software
